# Association of Current Active Illnesses and Severe Acute Kidney Injury after COVID-19 Vaccines: A Real-World Study

**DOI:** 10.3390/vaccines10050706

**Published:** 2022-04-29

**Authors:** Gang Chen, Qidong Ren, Jiannan Zhou, Yangzhong Zhou, Huiting Luo, Yining Wang, Xiaolin Li, Bin Zhao, Xuemei Li

**Affiliations:** 1 Nephrology Department, Institute of Materia Medica, Peking Union Medical College Hospital, Peking Union Medical College, Chinese Academy of Medical Sciences, Beijing 100730, China; chengang@pumch.cn (G.C.); rqd14@mails.tsinghua.edu.cn (Q.R.); jessica5322@sina.com (J.Z.); zhouyangzhong@pumch.cn (Y.Z.); pumc_luohuiting@student.pumc.edu.cn (H.L.); pumc_yining@student.pumc.edu.cn (Y.W.); 2 School of Medicine, Tsinghua University, Beijing 100730, China; 3 Nephrology Department, Beijing Aerospace General Hospital, Beijing 100076, China; 4 Pharmacy Department, Institute of Materia Medica, Peking Union Medical College Hospital, Peking Union Medical College, Chinese Academy of Medical Sciences, Beijing 100730, China; lixiaolin@imm.ac.cn (X.L.); zhaobin@pumch.cn (B.Z.); 5 State Key Laboratory of Bioactive Substance and Function of Natural Medicines, Institute of Materia Medica, Peking Union Medical College Hospital, Peking Union Medical College, Chinese Academy of Medical Sciences, Beijing 100730, China

**Keywords:** acute kidney injury, COVID-19, vaccines, adverse events following immunization, Vaccine Adverse Event Reporting System

## Abstract

The administration of COVID-19 vaccines has become increasingly essential to curb the pandemic. However, adverse events of acute kidney injury (AKI) emerge rapidly as the COVID-19 vaccination promotes. To investigate the intervenable risk factors of AKI, we searched the Vaccine Adverse Event Reporting System database and recorded adverse effects after COVID-19 vaccines from Dec 2020 to Jun 2021. We included 1149 AKI cases, of which 627 (54.6%) cases were reported following the Pfizer-BNT COVID-19 vaccine, and 433 (37.7%) were reported after the Moderna vaccine. A univariate analysis revealed that coexisting active illnesses (infections, uncontrolled hypertension, heart failure, etc.) have an unfavorable prognosis, with an increased risk of death (OR 2.35, 95% CI 1.70–3.25, *p* < 0.001). The other risk factors included older age and past disease histories. An adjusted regression analysis proved that coexisting active illnesses worsen AKI prognosis after COVID-19 vaccination, with a higher mortality risk (OR 2.19, 95% CI 1.48–3.25, *p* < 0.001). In subgroup analysis, we stratified different variables, and none revealed a significant effect modification on the association between coexisting active illnesses and AKI-associated death after vaccination (*p*-interaction >0.05). We found that coexisting active illnesses could complicate AKI after vaccines, but the potential causal relationship needed further investigation.

## 1. Introduction

Globally, as of 17th September 2021, more than 226 million confirmed coronavirus disease 2019 (COVID-19) cases, including 4.5 million deaths, have been reported to the World Health Organization (WHO) [1]. The rapid and mass application of COVID-19 vaccines has been one of the key strategies to curb the pandemic. The US Food and Drug Administration (FDA) issued two messenger RNA COVID-19 vaccines (Pfizer-BioNTech, USA and Germany, and Moderna, USA) in December 2020, which became two mainstream vaccines administered in the US [2,3]. A third adenovirus vector vaccine (Janssen) followed, and was approved in February 2021. As of mid-September 2021, more than 5.6 billion vaccine doses have been administrated globally, including over 379 million doses administrated in the United States (US) [1].

However, along with this massive-scale vaccination, acute kidney injury (AKI) cases were reported after COVID-19 vaccination [4,5,6,7,8,9,10,11]. Although the onset, clinical course, and prognosis of each case were not precisely the same, the emerging adverse events following immunization (AEFIs) of AKI draw our attention [4,5,6,7,8,9,10,11]. Various renal diseases accompanied with AKI were observed after vaccination, including IgA nephritis [4], minimal change diseases [5,6], membranous nephropathy [7], ANCA-associated vasculitis [8,9], anti-GBM nephritis [10], and renal thrombotic microangiopathy [11]. Patients with AKI tend to have worse outcomes, including the long-term development of chronic kidney disease, end-stage renal disease, and high mortality [12,13,14,15]. Considering the pandemic and its mortality risk, unlike other vaccines, COVID-19 vaccines have gradually become recommended to most of the population worldwide. This might potentially increase the incidence of AKI after COVID-19 vaccination.

We considered investigating AKI after COVID-19 vaccines in a larger population, rather than merely case reports. The pharmacovigilance of COVID-19 vaccines will be essential to determine the incidence of this potential adverse event (AE). We analyzed adverse effects reported to the Vaccine Adverse Event Reporting System (VAERS) from Dec 2020 to Jun 2021 to investigate the risk factors that could intervene to improve prognosis.

## 2. Methods

### 2.1. Database Description and Study Population

The VAERS database (https://vaers.hhs.gov, accessed date 5 July 2021) is a self-reporting system for post-vaccination AEs, jointly administered by the Centers for Disease Control and Prevention (CDC) and the US Food and Drug Administration (FDA). VAERS functions as an early warning system to detect possible safety issues with US-licensed vaccines, by collecting information about AEs after vaccination.

VAERS data contain the following files: VAERSDATA.CSV (including reports and patient information), VAERSVAX.CSV (including vaccine information), and VAERSSYMPTOMS.CSV (including the AE information). Patient information includes demographic data, coexisting active illnesses, past histories, allergy histories, and concurrent medications. Vaccine information includes vaccine manufacturers. AE information includes vaccination date, symptom onset date, AE description, and prognosis. We defined the time to AKI onset by calculating the interval between VAX_DATE (vaccination date) and ONSET_DATE (symptom onset date). 

We described all AKI reports to VAERS submitted for persons vaccinated with COVID-19 vaccines from December 2020 to June 2021. The records with important missing data (unclear prognosis after vaccine-associated AKI) were excluded. The records with incorrect entry or erred input (ONSET_DATE earlier than VAX_DATE) were excluded.

### 2.2. Outcome Measurement

The outcome measure targeted death associated with AKI after the COVID-19 vaccines. We used the MedDRA Version 23.1 for AKI mapping at the preferred term (PT) level. We considered the following PTs as related to AKI: “acute kidney injury [10069339]”, “subacute kidney injury [10081980]”, “blood creatinine increased [10005483]”, “blood urea abnormal [10005846]”, “glomerular filtration rate decreased [10018358]”, “renal impairment [10062237]”, “oliguria [10030302]”, “anuria [10002847]”, “dialysis [10061105]”, “renal tubular injury [10078933]”, “nephropathy toxic [10029155]”, “nephritis allergic [10029120]”, and “tubulointerstitial nephritis [10048302]”. We used IBM Micromedex as a dictionary to select the brand names for the COVID-19 vaccines.

In this study, we used a similar approach to the previous analysis of the VAERS database to ensure the proper interpretation of natural language and consistency of evaluation methods [16]. Two physicians who specialized in nephrology, with more than seven years of experience independently, analyzed the descriptions in the database to ensure the reliability of the AKI diagnosis (excluding the maintenance dialysis patient at baseline, etc.). They independently analyzed coexisting active illnesses, past histories, allergies, and concurrent medications described in natural language in VAERS. If they disagreed with the judgment of the description, they would turn to a professor with more than 20 years of experience in nephrology. In interpreting the database, the coexisting active illnesses included current active infections (pneumonia, urinary tract infection, upper respiratory infection, and other infections), uncontrolled heart failure (described as heart failure when vaccinated in VAERSDATA.CSV), uncontrolled hypertension (described as hypertension not well-controlled when vaccinated in VAERSDATA.CSV), active gastrointestinal disorders (vomiting and diarrhea), active autoimmune diseases, active glomerulopathy, and recent bone fracture or joint diseases.

### 2.3. Statistical Analysis

We conducted a descriptive analysis and compared demographic data, coexisting active illnesses, past histories, allergy histories, and concurrent medications between survived and non-survived AKI cases. Continuous data were presented as mean ± standard deviation and compared by *t*-test or Mann-Whitney *U* test. Categorical data were presented as percentages and compared by Pearson chi-square test or Fisher’s exact test. We first examined the differences between deceased AKI patients following vaccination and surviving AKI vaccinees. Univariate regression analysis was applied to estimate the effect size between all variates between the two groups. Multivariable analyses were used to evaluate the associations between the coexisting active diseases and death after vaccine-associated AKI in three models: model I (not adjusted for other covariates), model II (adjusted for age and gender), and model III (adjusted for the same factors as those in model II, and other associated factors in univariate analysis). We performed a variance inflation factors (VIFs) test to screen the collinearity of the variates. We also explored the relationship between coexisting active illnesses and death after vaccine-associated AKI in subgroups, to detect potential interactions. Differences were considered significant if *p* < 0.05. Statistical analysis was performed by SPSS, version 21.0 (IBM Inc., Armonk, NC, USA). 

## 3. Results

### 3.1. Baseline Characteristics of Selected Patients

**Figure 1:**
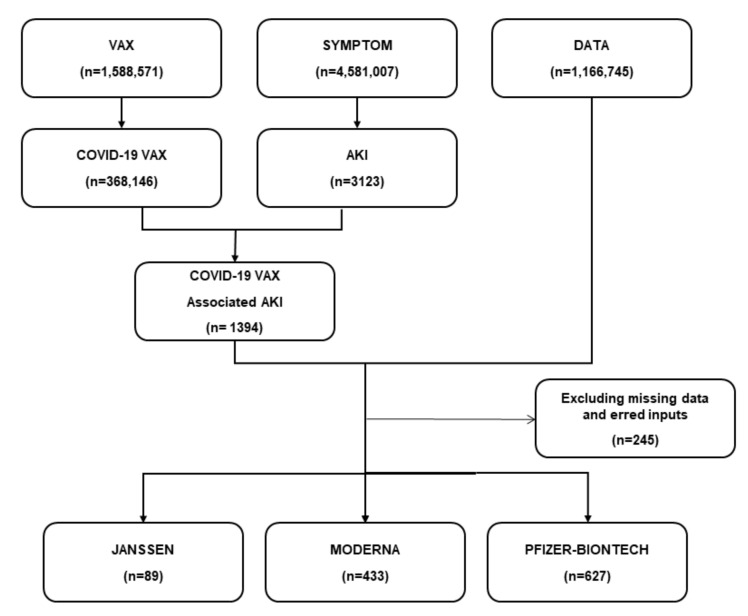
The flow diagram of the study. A total of 1394 AKI cases were found after the administration of the COVID-19 vaccine. After combining with patient data and excluding missing data and erred inputs, 1149 AKI cases were finally selected in this study. Of these, 627 (54.6%) cases were reported following the Pfizer-BNT COVID-19 vaccine, and 433 (37.7%) were reported after the Moderna vaccine.

From December 2020 to June 2021, 368,146 AEs related to COVID-19 vaccines were documented in the VAERS database. We screened 1394 AKI cases after the administration of the COVID-19 vaccine. After combining with patient data, and excluding missing data and erred inputs, we finally included 1149 AKI cases in this study. The survived group had 937 (81.55%) cases, and the non-survived group had 212 (18.45%) cases. Among all AKI cases, 627 (54.6%) cases were reported following the Pfizer-BNT COVID-19 vaccine, and 433 (37.7%) were reported after the Moderna vaccine (Figure 1).

Table 1 shows the baseline characteristics of the survived and non-survived AKI cases after COVID-19 vaccines. The baseline characteristics were markedly imbalanced between the two groups. The non-survived AKI patients (75.45 ± 12.81 years) were older than the survived patients (65.37 ± 17.22 years) (*p* < 0.001). The status of coexisting active illnesses at the time of vaccination was significantly higher in the non-survived patients than in the survived group (35.85% vs. 19.53%, *p* < 0.001), especially infections (13.21% vs. 5.23%, *p* < 0.001) and uncontrolled heart failure (4.25% vs. 1.71%, *p* < 0.001). Past disease histories were also more dominant in the non-survived group than in the survived group (66.35% vs. 57.20%, *p* < 0.001). Compared with the survived patients, the non-survived patients were documented as having more past histories of heart diseases (8.43% vs. 10.90%, *p* < 0.001), gastrointestinal diseases (13.87% vs. 22.27%, *p* = 0.002), and chronic kidney diseases (19.53% vs. 21.80%, *p* = 0.049). There was no difference in allergy histories and meantime medicine usage between the two groups. The average time to AKI onset was 12.59 ± 18.39 days in all cases, and there was no difference between the two groups. In the non-survived group, the death from AKI onset was averaged to 8.56 ± 13.89 days. The two groups did not differ in various documented AKI causes, including volume depletion, sepsis, acute tubular necrosis, acute interstitial nephritis, glomerular nephritis, etc. Unexpectedly, clinic visits (14.15% vs. 21.88%, *p* = 0.012), emergency visits (47.17% vs. 55.92%, *p* = 0.021), and hospitalizations (55.66% vs. 74.92%, *p* < 0.001) were lower in the non-survived AKI patients, compared with the survived patients.

### 3.2. Univariate Analysis

We performed a univariate analysis to evaluate the risk factors of AKI-associated mortality following immunization (Table 2). The analysis revealed that coexisting active illnesses have an unfavorable prognosis with an increased risk in death (OR = 2.35, 95% CI = 1.70–3.25, *p* < 0.001). The other factors, including older age and past disease histories, were associated with increased AKI-associated mortality, whereas emergency visits and hospitalizations contributed to a decreased AKI-associated mortality.

### 3.3. Unadjusted and Adjusted Regression Analysis Models

We constructed three models to analyze the independent effect of coexisting active illnesses on AKI prognosis after COVID-19 vaccination (Table 3). In the unadjusted crude model (model I), the AKI-associated mortality was 2.35 times higher in vaccinees with coexisting active illnesses (OR = 2.35, 95% CI = 1.70–3.25, *p* < 0.001). In the minimum-adjusted model (Model II, adjusted age and gender), the AKI-associated mortality was 2.42 times higher in vaccinees with coexisting active illnesses (OR = 2.42, 95% CI = 1.71–3.45, *p* < 0.001). We performed a VIFs test for the variates in Table 2, and all the values were less than 5. Then we fully adjusted Model III (adjusted covariates provided in Table 2), which indicated that AKI caused up to a 2.19-fold increased risk in death in vaccinees with coexisting active illnesses compared with patients without coexisting active illnesses (OR = 2.19, 95% CI = 1.48–3.25, *p* < 0.001).

### 3.4. Subgroup Analysis

We conducted a subgroup analysis to verify the robustness of the findings across the potential confounders. We stratified age (<64, 64–75, and >75 years), gender, past disease histories, chronic kidney diseases, allergy histories, clinic visits, emergency visits, hospitalization, and dialysis treatment (Table 4). None of the variables revealed a significant effect modification of the association between coexisting active illnesses and AKI-associated death after vaccination (*p*-interaction > 0.05).

## 4. Discussion

The current study showed that, after receiving COVID-19 mRNA vaccination, coexisting active illnesses had a higher risk of AKI-associated mortality than those without coexisting active illnesses. The model effect showed that AKI-associated mortality was 2.19 times higher in vaccinees with coexisting active illnesses than in those without active illnesses. A subgroup analysis robustly revealed that coexisting active illnesses at the time of vaccination could serve as an independent prognostic indicator in patients who developed AKI after the COVID-19 vaccines.

We previously reported that the COVID-19 vaccines issued in the US are generally safe in the short term with non-severe AEFIs [16]. Among all 3908 AEFIs reported to VAERS until January 2021, general disorders, such as fatigue, pain, and chills, were the most common (48.80%), followed by nervous system disorders (46.39%) [16]. In contrast, renal and urinary disorders were among the least-reported AEFIs (0.46%) [16]. However, after several months of a rapid increase in vaccination, such rare but important AEFIs have accumulated.

AKI cases were previously reported after other vaccines, including the influenza vaccine [17], yellow fever vaccine [18], and typhoid and cholera vaccines [19]. However, AKI reports following the COVID-19 vaccination were more frequent throughout the literature search than for other vaccines. Though the large scale of COVID-19 vaccination could partially and briefly explain the emerging AKI cases, several other factors may account for AKI following COVID-19 vaccines. First, the high immunogenicity, especially featured in the mRNA vaccines, might act as a double-edged sword. Renal AEs were more commonly reported in mRNA COVID-19 vaccines than in other types [20]. It was also a fact that the majority of AKI cases in this study were received with mRNA vaccines. After almost two decades of refinement, mRNA vaccines can induce robust cell-mediated and antibody-medicated immune responses [21]. Clinical trials and real-world observations also revealed their superiority over traditional vaccines [22,23,24,25]. However, concerns arose that unexpected and non-specific immune activation may be triggered, aggravated, or incite autoimmune activities [20]. The kidney could be insulted during such processes, which occur in AKI [4,5,6,7,8,9,10,11]. Second, the immune response to COVID-19 vaccines may mimic a response to natural infection, thus resulting in AKI. Our previous meta-analysis estimated that AKI prevalence during the natural COVID-19 process was 11.46% in a collection of 8735 patients [26]. The symptoms of some AEs after vaccines could also be seen in the natural course of COVID-19, though they were far rarer and milder [16]. AKI may be motivated by the same scenario. It should be noted that the VAERS database was not suitable for setting up a causal relationship, and some of the causes of AKI in our analysis may not be directly driven by vaccination. Still, the database was of great importance as a hypothesis-generating system with the primary goal of detecting safety signals that might be related to vaccination.

Our study deduced that coexisting active illnesses worsened the prognosis of AKI following the COVID-19 vaccines. In general, people with active diseases are routinely excluded from vaccination. However, due to the severity and rapid spread of COVID-19, persons with non-incumbent diseases may thus be vaccinated when the potential benefits of vaccination are more highly valued. We could identify some latent risks among the entries for the active diseases analyzed in this study. For instance, unrecovered infections or active autoimmune disorders may interrupt vaccine-induced immune responses and lead to enhanced kidney damage; uncontrolled heart failure, hypertension, vomiting, and diarrhea could result in volume depletion or decreased renal perfusion. On the other hand, if AKI occurs in patients with an active disease, AKI and the existing active disease may interact to exacerbate the extent of AKI. AKI is already a severe medical scenario associated with an in-hospital mortality of 11% [27]; this mortality rate soars to 45–60% when AKI is complicated by other organ interactions, such as pneumonia, acute heart failure, or sepsis [28,29]. Recent clinical and basic research demonstrated that AKI induces systemic and organ-specific hemodynamic, humoral, and immunologic imbalances [29], thus complicating the clinical condition and worsening the prognosis if the patients are already suffering from active illnesses of other organs. Due to the potential worsening consequences of AKI following vaccines, we strongly recommend vaccination be withheld for patients who still have active diseases.

We admit that there are some limitations to our study. First, unlike researchers in clinical trials who report AEs using standardized data collection methods, the quality of information submitted by VAERS reporters varies widely. We interpreted the description in the database with scrutiny by experienced nephrologists and cleaned the data into analyzable forms, but the effort did not work for some vague descriptions. Second, as a passive spontaneous reporting system, VAERS is inhibited with reporting biases, overreporting or underreporting, and a lack of an unvaccinated comparison group. Although it is less likely to under-report a concerning AE, some mild or moderate AEs may be more frequent than we assessed in the database. Third, VAERS does not provide denominator data to calculate the incidence rates of AEs; therefore, we could not calculate accurate incidence rates by interpreting the VAERS database. Fourth, the analysis of the VAERS database can only reveal clinical correlations but not causality.

## 5. Conclusions

AKI is a rare but potentially severe AEFI following COVID-19 vaccination. We confirmed in this study that coexisting active illnesses would complicate the condition if AKI occurred after the vaccines. Although this study cannot determine a causal relationship between the vaccine and deteriorated AKI in patients with active illnesses, ongoing surveillance for similar complications is prudent as our worldwide vaccination efforts continue. We recommend assessing the detailed profiles of vaccine recipients who develop severe AEFIs to explore the potential causal relationship between clinical characteristics and AKI following vaccines.

## Figures and Tables

**Table 1:** Clinical characteristics in survived AKI patients after COVID-19 vaccination compared to deceased patients, sourced from the VAERS database (December 2020 to June 2021).

Clinical Characteristics	Total (*n* = 1149)	Survived (*n* = 937)	Non-Survived (*n* = 212)	*p*
Demographics				
Age, years	67.16 ± 16.97	65.37 ± 17.22	75.45 ± 12.81	<0.001 *
Male, *n* (%)	590 (51.62)	471 (50.59)	119 (56.13)	0.145
Coexisting active illnesses				
Total, *n* (%)	256 (22.28)	180 (19.21)	76 (35.85)	<0.001 *
Infection, *n* (%)	81 (7.05)	51 (5.44)	30 (14.15)	<0.001 *
Pneumonia, *n* (%)	38 (3.31)	21 (2.24)	17 (8.02)	<0.001 *
Urinary tract infection, *n* (%)	17 (1.48)	13 (1.39)	4 (1.89)	0.587
Upper respiratory tract infection, *n* (%)	3 (0.26)	2 (0.21)	1 (0.47)	0.458
Other infection, *n* (%)	26 (2.26)	16 (1.71)	10 (4.72)	0.008 *
Heart failure, *n* (%)	24 (2.09)	13 (1.39)	11 (5.19)	<0.001 *
Uncontrolled hypertension, *n* (%)	40 (3.51)	22 (2.36)	18 (8.65)	<0.001 *
GI conditions (vomiting, diarrhea or bleeding), *n* (%)	16 (1.40)	12 (1.29)	4 (1.92)	0.482
Active glomerulonephritis or immune diseases, *n* (%)	20 (1.76)	16 (1.72)	4 (1.92)	0.839
Recent fracture or arthralgia, *n* (%)	19 (1.67)	9 (0.97)	10 (4.81)	<0.001 *
Other active illnesses, *n* (%)	89 (7.75)	68 (7.26)	21 (9.91)	0.193
Past disease histories				
Total, *n* (%)	676 (58.89)	536 (57.20)	140 (66.35)	0.015 *
Hypertension, *n* (%)	355 (30.92)	278 (29.67)	77 (36.49)	0.053
Diabetes, *n* (%)	226 (19.70)	180 (19.23)	46 (21.80)	0.396
Chronic kidney diseases, *n* (%)	237 (20.64)	183 (19.53)	54 (25.59)	0.049 *
Heart diseases, *n* (%)	235 (20.47)	172 (18.36)	63 (29.86)	<0.001 *
Asthma and COPD, *n* (%)	102 (8.89)	79 (8.43)	23 (10.90)	0.255
Gastrointestinal diseases, *n* (%)	177 (15.42)	130 (13.87)	47 (22.27)	0.002 *
Connective tissue diseases, *n* (%)	31 (2.70)	28 (2.99)	3 (1.42)	0.205
Anemia, *n* (%)	54 (4.70)	39 (4.16)	15 (7.11)	0.068
Allergy histories, *n* (%)	380 (33.07)	309 (32.98)	71 (33.49)	0.886
Meantime medicine usage				
Antihypertensives	384 (33.42)	323 (34.47)	61 (28.77)	0.112
RAS inhibitors	193 (16.80)	160 (17.08)	33 (15.57)	0.595
Diabetes medicines	149 (12.97)	124 (13.23)	25 (11.79)	0.573
Cardiovascular medicines	62 (5.40)	53 (5.66)	9 (4.25)	0.412
Steroids	33 (2.87)	25 (2.67)	8 (3.77)	0.384
Immunosuppressives	31 (2.70)	25 (2.67)	6 (2.83)	0.895
Documented AKI causes				
Volume depletion, *n* (%)	435 (37.86)	360 (38.42)	75 (35.38)	0.409
Nausea and vomiting, *n* (%)	211 (18.36)	176 (18.78)	35 (16.51)	0.440
Diarrhea, *n* (%)	111 (9.66)	93 (9.93)	18 (8.49)	0.523
Fever, *n* (%)	293 (25.50)	244 (26.04)	49 (23.11)	0.377
Decreased appetite, *n* (%)	35 (3.05)	31 (3.31)	4 (1.89)	0.277
Sepsis, *n* (%)	133 (11.58)	105 (11.21)	28 (13.21)	0.411
Acute tubular necrosis, *n* (%)	12 (1.04)	8 (0.85)	4 (1.89)	0.182
Acute interstitial nephritis, *n* (%)	2 (0.17)	2 (0.21)	0 (0.00)	0.501
Glomerular nephritis, *n* (%)	22 (1.91)	18 (1.92)	4 (1.89)	0.974
Nephrotic syndrome, *n* (%)	2 (0.17)	2 (0.21)	0 (0.00)	0.501
Thrombotic microangiopathy, *n* (%)	53 (4.61)	45 (4.80)	8 (3.77)	0.519
Medical records				
Time to AKI onset, days	12.59 ± 18.39	12.41 ± 18.43	13.39 ± 18.22	0.488
Clinic visit after AKI, *n* (%)	235 (20.45)	205 (21.88)	30 (14.15)	0.012 *
ER visit after AKI, *n* (%)	624 (54.31)	524 (55.92)	100 (47.17)	0.021 *
Hospitalization for AKI, *n* (%)	820 (71.37)	702 (74.92)	118 (55.66)	<0.001 *
Length of stay, days	3.71 ± 5.77	3.82 ± 5.54	3.30 ± 6.59	0.307
Dialysis initiated, *n* (%)	100 (8.70)	82 (8.75)	18 (8.49)	0.903

* Significance was found (*p* < 0.05). Abbreviations: COVID-19: coronavirus disease 19; AKI: acute kidney injury; VARES: Vaccine Adverse Event Reporting System; GI: gastrointestinal; RAS: renin–angiotensin system; ER: emergency room.

**Table 2:** Univariate analysis for deceased AKI patients after COVID-19 vaccine.

	No. of Participants, *n* (%)	OR (95% CI)	*p*
Gender, *n* (%)			
Male	590 (51.62)	1.0	
Female	553 (48.38)	0.80 (0.59, 1.08)	0.146
AGE group, *n* (%)			
<64 years	366 (32.85)	1.0	
64–75 years	358 (32.14)	2.14 (1.32, 3.47)	0.002
>75 years	390 (35.01)	5.05 (3.24, 7.86)	<0.001
AGE group trend	67.75 ± 14.50	1.05 (1.03, 1.06)	<0.001
Coexisting active illnesses, *n* (%)			
No	893 (77.72)	1.0	
Yes	256 (22.28)	2.35 (1.70, 3.25)	<0.001
Past disease histories, *n* (%)			
No	472 (41.11)	1.0	
Yes	676 (58.89)	1.48 (1.08, 2.02)	0.015
Chronic kidney diseases, *n* (%)			
No	911 (79.36)	1.0	
Yes	237 (20.64)	1.42 (1.00, 2.01)	0.050
Allergy histories, *n* (%)			
No	769 (66.93)	1.0	
Yes	380 (33.07)	1.02 (0.75, 1.40)	0.886
Meantime anti-hypertensives usage, *n* (%)			
No	765 (66.58)	1.0	
Yes	384 (33.42)	0.77 (0.55, 1.06)	0.113
Meantime RAS inhibitors usage, *n* (%)			
No	956 (83.20)	1.0	
Yes	193 (16.80)	0.90 (0.60, 1.35)	0.596
Clinic visit after AKI, *n* (%)			
No	914 (79.55%)	1.0	
Yes	235 (20.45%)	0.59 (0.39, 0.89)	0.013
ER visit after AKI, *n* (%)			
No	525 (45.69)	1.0	
Yes	624 (54.31%)	0.70 (0.52, 0.95)	0.021
Hospitalization, *n* (%)			
No	329 (28.63)	1.0	
Yes	820 (71.37)	0.42 (0.31, 0.57)	<0.001
Dialysis initiated after AKI, *n* (%)			
No	1049 (91.30)	1.0	
Yes	100 (8.70)	0.97 (0.57, 1.65)	0.903

Abbreviations: COVID-19: coronavirus disease 19; AKI: acute kidney injury; CI: confidential interval; OR: odds ration; RAS: renin-angiotensin system; ER: emergency room.

**Table 3:** Risk association of coexisting active illnesses in vaccinees and AKI death in different models.

Exposure	Model I (Crude) OR (95% CI), *p*-Value	Model II OR (95% CI), *p*-Value	Model III OR (95% CI), *p*-Value
Coexisting active illnesses			
No	1.0	1.0	1.0
Yes	2.35 (1.70, 3.25), *p* < 0.001	2.42 (1.71, 3.42), *p* < 0.001	2.19 (1.48, 3.25), *p* < 0.001

Abbreviations: CI: confidential interval; OR: odds ratio. Crude model, adjusted for none. Model II adjusted for age and gender. Model III adjusted for age, gender, past disease histories, allergy histories, CKD histories, meantime antihypertensives usage, meantime RAS inhibitors usage, clinic visits after AKI, ER visits after AKI, hospitalizations, and dialysis initiated after AKI. Abbreviations: AKI: acute kidney injury; CI: confidential interval; OR: odds ratio; RAS: renin–angiotensin system; ER: emergency room.

**Table 4:** The effect size on deceased AKI patients after COVID-19 vaccines and exploratory subgroups.

Tercile of Coexisting Active Illnesses	No of Participants	Effect Size OR (95% CI)	*p* for Interaction
Age group, years			0.354
<64 years	366	1.56 (0.62, 3.93)	
64–75 years	358	3.42 (1.66, 7.04)	
>75 years	390	2.00 (1.10, 3.61)	
Gender			0.389
Male	590	2.71 (1.54, 4.75)	
Female	553	1.90 (1.07, 3.39)	
Past disease histories			0.272
No	472	1.18 (0.34, 4.05)	
Yes	676	2.41 (1.57, 3.69)	
Chronic kidney diseases			0.291
No	911	1.93 (1.22, 3.07)	
Yes	237	3.17 (1.43, 7.03)	
Allergy histories			0.079
No	769	1.63 (0.93, 2.86)	
Yes	380	3.37 (1.88, 6.04)	
Clinic visit after AKI			0.475
No	914	2.40 (1.57, 3.65)	
Yes	235	1.45 (0.38, 5.47)	
ER visit after AKI			0.442
No	525	2.66 (1.53, 4.61)	
Yes	624	1.91 (1.02, 3.60)	
Hospitalization			0.260
No	329	1.57 (0.79, 3.11)	
Yes	820	2.54 (1.54, 4.20)	
Dialysis initiated after AKI			0.350
No	1049	2.07 (1.36, 3.14)	
Yes	100	3.93 (1.10, 14.03)	

Abbreviations: COVID-19: coronavirus disease 19; AKI: acute kidney injury; CI: confidential interval; OR: odds ration; RAS: renin–angiotensin system; ER: emergency room.

## Data Availability

All necessary data are presented as tables and figures in the manuscript. Related information is accessible under request to the corresponding author.

## Abbreviations

COVID-19: coronavirus disease 19; WHO: World Health Organization; AKI: acute kidney injury; AE: adverse event; AEFI: adverse event following immunization; VAERS: Vaccine Adverse Event Reporting System; FDA: Food and Drug Administration; CDC: Centers for Disease Control and Prevention; MedDRA: Medical Dictionary for Regulatory Activities.
